# Safety and Efficacy of Obinutuzumab in Treating Lupus Nephritis: A Systematic Review

**DOI:** 10.1002/iid3.70439

**Published:** 2026-04-13

**Authors:** Zainab Arif, Hafsa Ali, Erum Siddiqui, Shorrem Naeem, Mohammed Hammad Jaber Amin

**Affiliations:** ^1^ Faculty of Medicine, Sindh Medical College Jinnah Sindh Medical University Sindh Karachi Pakistan; ^2^ Faculty of Medicine Alzaiem Alazhari University Khartoum North Khartoum Sudan

**Keywords:** adverse events, B‐cell depletion, complete renal response, lupus nephritis, obinutuzumab, proteinuria, randomized controlled trials, systemic lupus erythematosus

## Abstract

**Background:**

Lupus nephritis (LN) is a serious manifestation of systemic lupus erythematosus (SLE), often leading to end‐stage renal disease. Despite current immunosuppressive therapies, complete renal remission remains limited. Obinutuzumab, a type II anti‐CD20 monoclonal antibody, offers enhanced B‐cell depletion compared to type I antibodies and may improve renal outcomes in LN. This systematic review evaluates the efficacy and safety of obinutuzumab in treating adult patients with proliferative LN.

**Methods:**

This review was registered with PROSPERO (CRD420251074418) and conducted in accordance with the PRISMA guidelines. A comprehensive search of PubMed, Cochrane, Embase, Scopus, and clinical trial registries was conducted through June 2025. Eligible studies were randomized controlled trials (RCTs) comparing obinutuzumab vs. placebo in adult patients with biopsy‐confirmed active LN. Primary outcomes included complete renal response (CRR), proteinuria, and estimated glomerular filtration rate (eGFR). Secondary outcomes included immunological markers and adverse events. Data were synthesized qualitatively due to the limited available trials.

**Results:**

Two RCTs (REGENCY 2025 and NOBILITY 2022) with 396 patients met the inclusion criteria. Obinutuzumab significantly improved CRR (46.4% in REGENCY at week 76; 41% in NOBILITY at week 104) and reduced proteinuria (UPCR < 0.5 in 62% vs. 37% in controls). Improvements in eGFR, complement levels (C3 and C4), and anti‐dsDNA titers were also noted. Adverse events occurred at similar rates across groups, although serious infections and infusion reactions were somewhat higher in one trial. No significant increase in mortality was observed.

**Conclusion:**

Obinutuzumab appears to be a promising therapy for proliferative LN, offering enhanced renal response and immunological benefits. Its potential for long‐term renal protection and steroid‐sparing effects supports further investigation through large‐scale comparative trials.

## 1 Introduction

Lupus nephritis (LN) is a disorder characterized by immune‐mediated glomerulonephritis, which is the most severe and commonly occurring complication of systemic lupus erythematosus (SLE), an autoimmune disease involving several organ systems at once or successively [1, 2]. The renal system is most commonly affected by SLE, derived from glomerular, tubular, and vascular lesions [3]. It occurs in 40% of SLE patients and is more prevalent in females, with a female‐to‐male ratio of approximately 1:5 [2, 4]. The American College of Rheumatology (ACR) classifies LN as proteinuria exceeding 0.5 g/day or a urinary protein/creatinine ratio (UPCR) beyond 0.5 or urinary protein of more than 3+ using a urinary dipstick or greater than five cells per high‐power field of urinary cellular casts [5]. The 2025 American College of Rheumatology (ACR) guidelines recommend early diagnosis and stratified treatment based on disease class and activity, with renal biopsy remaining the gold standard for confirming LN and guiding therapy [6]. The clinical presentation of LN is quite heterogeneous, ranging from undetectable urinary anomalies to distinctive cases of nephrotic syndrome, microscopic hematuria, proteinuria, decreased glomerular filtration rate (GFR), and rapidly advancing renal insufficiency [2, 3].

The preferred diagnostic method involves collecting urine over 24 h to observe protein excretion. This can be made more robust by simultaneously measuring creatinine levels in the urine to account for changes in protein excretion caused by physical activity or collection mistakes. Sediments in urine are also helpful in determining renal disease, as the presence of hemoglobin, leukocytes, and casts is characteristic of the disease. For histological identification and precise diagnosis of LN, kidney biopsy is the gold standard [3].

LN's therapeutic plan comprises induction and maintenance stages. The induction phase consists of high‐dose GCs integrated with cyclophosphamide (CYC) or mycophenolate mofetil (MMF) to induce remission of the active condition. Low‐dose GCs combined with MMF or azathioprine (AZA) are employed in the maintenance phase for sustained remission [1, 4]. However, current therapies do not have satisfactory efficacy in inducing remission and preventing new disease outbursts, and not all patients show a good prognosis. Less than 30% of the individuals attain complete remission in 6 months of therapy. Eventually, up to 20% of the individuals with LN end up developing end‐stage renal disease [7]. Biologic therapies, including rituximab, a type I anti‐CD20 monoclonal antibody, and anifrolumab, an anti‐interferon receptor antibody, have shown promise in SLE and LN, particularly for patients with refractory disease or those at high risk of organ damage. Despite these advances, a significant proportion of patients fail to achieve sustained renal remission, highlighting the need for novel therapies, such as obinutuzumab, that may offer improved efficacy and immunologic modulation in LN management [8].

Obinutuzumab is a humanized type 2 anti‐CD20 monoclonal antibody that has shown considerably improved renal outcomes compared to placebo in patients with LN. It has previously been approved for treating follicular lymphoma and chronic lymphocytic leukemia and has shown more affinity than rituximab for treating both diseases [9]. B cells are the principal mediators of SLE pathogenesis; however, type 1 anti‐CD20 antibodies like rituximab and ocrelizumab show inconsistent B‐cell depletion, hence exhibiting low rates of complete renal response (CRR). This resistance in reducing B‐cells by type 1 anti‐CD20 antibodies may result from the internalization of CD20 mediated by Fcγ receptor IIB (FcγRIII), inefficient complement‐dependent cytotoxicity, and deficient phagocytosis. Obinutuzumab binds to the CD20 antigen and has a greater affinity for the Fcγ FcγRIII on effector cells than the type I anti‐CD20 antibodies. This leads to direct B‐cell death, better antibody‐reliant cellular cytotoxicity, and reduced dependency on complement‐dependent cytotoxicity compared to type I anti‐CD20 antibodies [10].

Preliminary clinical data indicate that obinutuzumab may improve renal outcomes in LN patients and delay disease flares more effectively than existing therapies [9]. Given these promising yet emerging results, this systematic review comprehensively assessed obinutuzumab's safety and efficacy in treating LN. This review will help consolidate current evidence, clarify its comparative advantages, and guide future therapeutic decision‐making in managing this challenging condition.

## 2 Methodology

This systematic review was registered with PROSPERO 2025 CRD420251074418, Available from https://www.crd.york.ac.uk/PROSPERO/view/CRD420251074418 and carried out in compliance with the Preferred Reporting Items for Systematic Reviews and Meta‐Analyses (PRISMA) guidelines.

## 3 Literature Search

This meta‐analysis was conducted conclusively with the Preferred Items for Systematic Reviews and Meta‐Analysis (PRISMA) guidelines [11]. The following search terms were used from inception to June 2025: (“Lupus Nephritis” OR “proliferative lupus nephritis” OR “lupus glomerulonephritis”) AND (“Obinutuzumab” OR “GA101” OR “type II anti‐CD20”). A thorough electronic search was performed on PubMed (Medline), Cochrane Central, Scopus, Embase, and other focused searches. The reference lists of the included studies, relevant meta‐analyses and review papers, presentations at international conferences, and the clinicaltrials.gov website were all consulted in the search for gray literature.

### 3.1 Study Selection and Eligibility Criteria

All randomized controlled trials (RCTs) that assessed obinutuzumab's safety and effectiveness in comparison to a placebo in adult patients with biopsy‐confirmed active lupus nephritis (LN) were included in this systematic review. At least one relevant clinical outcome, such as renal responsiveness, proteinuria levels, estimated glomerular filtration rate (eGFR), or treatment‐related side events, had to be reported by eligible trials.

Studies were only taken into consideration if they included subjects who were at least 18 years old and had a verified diagnosis of lupus nephritis according to accepted clinical and histological standards. There were no restrictions regarding the geographic location or language of publication, as long as the full‐text articles were accessible.

Studies were excluded if they: did not directly contrast obinutuzumab with a placebo, without particular information on lupus nephritis, the study concentrated on patients with systemic lupus erythematosus (SLE). Involved contrasting non‐placebo drugs (such as different monoclonal antibodies), weren't RCTs (e.g., single‐arm trials, case series, and observational studies), were non‐original works, such as abstracts from conferences, reviews, editorials, and commentaries. Two randomized controlled trials that satisfied the inclusion criteria were added to the final analysis after a rigorous screening process.

### 3.2 Data Extraction

Duplicate studies were eliminated from the list after exporting the acquired articles to Rayyan.ai. Two reviewers independently screened the abstracts and titles to exclude irrelevant research; only those that satisfied the previously mentioned eligibility requirements were included, and their complete texts were collected. To settle any disputes about the findings, a third author was consulted. One author used the data from the completed studies for the baseline characteristics to generate an online Microsoft Excel spreadsheet. The following are baseline parameters: eGFR mL/min/1.73 m^2^, C3 and C4 complement levels (mg/dL), serum creatinine, evaluated research arms, population characteristics, and total number of participants.

### 3.3 Risk of Bias Assessment

The Cochrane Risk of Bias 2 (RoB 2) tool [12] was used to assess the quality of included RCTs. A single reviewer (ZA) evaluated the risk of bias across the following domains: randomization process, deviations from intended interventions, missing outcome data, measurement of outcomes, and selection of reported results. Disagreements were resolved through discussion with a second party.

### 3.4 Primary Outcomes

Complete renal response, urinary protein creatinine ratio, and changes in estimated glomerular filtration rate.

### 3.5 Secondary Outcomes

Changes in C3 and C4 levels, anti‐dsDNA antibodies (immunological effects), renal and infection‐related adverse events, and mortality.

### 3.6 Data Synthesis

Conducting a meta‐analysis was not feasible due to the unavailability of sufficient data. While both studies reported primary and secondary outcomes, the number of studies retrieved was restricted to only two. This small sample size did not meet the methodological requirements for a robust and statistically meaningful meta‐analysis. Therefore, we conducted a systematic review of all published randomized controlled trials to qualitatively assess the existing evidence regarding the safety and efficacy of obinutuzumab in patients with lupus nephritis.

## 4 Results

### 4.1 Study Identification

**FIGURE 1:**
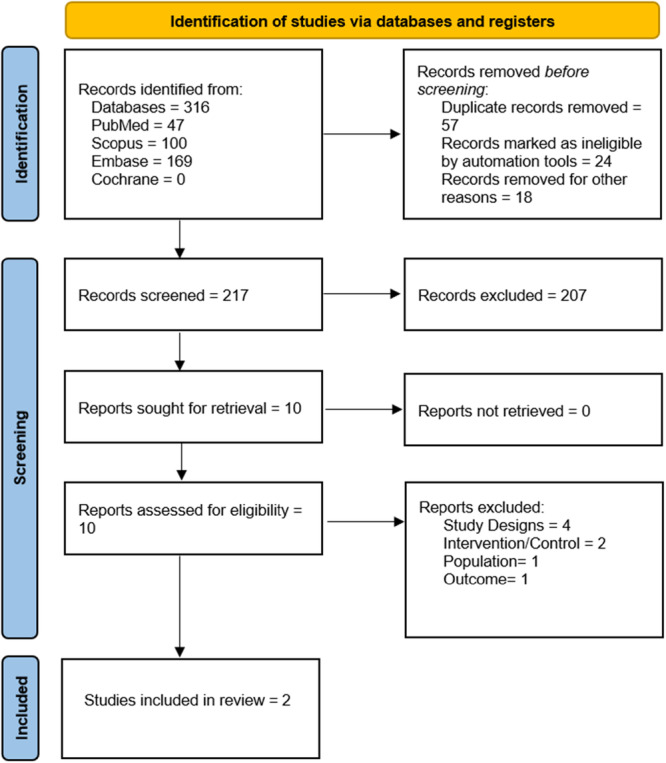
PRISMA flowchart.

A total of 316 articles were identified through various database searches. After removing 57 duplicate records and studies that were considered ineligible by automation tools, 217 articles remained for screening. Title and abstract screening led to the exclusion of 207 articles. The full texts of the remaining 10 articles were assessed for eligibility, of which eight were excluded for not meeting the inclusion criteria. Ultimately, two randomized controlled trials were included in the final analysis. The PRISMA flowchart is shown in Figure 1.

### 4.2 Characteristics of Studies

All of the included studies were randomized controlled trials that evaluated obinutuzumab in patients diagnosed with lupus nephritis. The trials evaluated the safety and efficacy of obinutuzumab in addition to standard therapy vs. a placebo with standard therapy. Adult patients who had biopsy‐confirmed active lupus nephritis—most frequently ISN/RPS Class III or IV—with or without Class V involvement made up the majority of the study groups. The majority of patients were on background immunosuppressive treatment, which was uniform across trial arms and typically included glucocorticoids and mycophenolate mofetil. Studies differed slightly in baseline renal and serologic markers, including eGFR, anti‐dsDNA levels, and urine protein: creatinine ratio (UPCR). Monitoring for infections, severe adverse events, and infusion responses was part of the safety data. Table 1a provides a breakdown of patient demographics, and Table 1b summarizes the baseline characteristics of the included studies.

**TABLE 1a:** Patient demographics.

Parameter	REGENCY 2025 (obinutuzumab/control)	NOBILITY 2022 (obinutuzumab/control)
Total participants (*n*)	135/136	63/62
Age (years, mean ± SD)	33.0 ± 10.5/32.7 ± 10.0	33.1 ± 9.8/31.9 ± 10.1
Female sex, *n*	114/115	55/51
Region (*n*)		
Latin America and Caribbean	Not reported	38/47
Europe and Israel	Not reported	18/7
USA	Not reported	7/8
Race (*n*)		
White, *n*	65/64	28/26
American Indian or Alaska Native, *n*	25/26	11/17
Black or African American, *n*	20/20	6/5
Asian, *n*	9/7	3/2
Other or unknown, *n*	19/19	15/12
Ethnicity (*n*)		
Hispanic or Latino, *n*	71/85	Not reported
Not Hispanic or Latino, *n*	54/48	Not reported
Other or unknown, *n*	10/3	Not reported

**TABLE 1b:** Baseline clinical and biochemical characteristics.

Category	Parameter	REGENCY 2025 (obinutuzumab/control)	NOBILITY 2022 (obinutuzumab/control)
Clinical	Prior history of lupus nephritis, *n*	81/76	32/32
Histological	Class IV lupus nephritis, *n*	Not reported	40/35
	Concomitant Class V lupus nephritis, *n*	Not reported	20/17
Biochemical	Serum creatinine (mg/dL)	0.79 (0.34–3.75)/0.74 (0.27–4.39)	0.87 ± 0.34/0.80 ± 0.33
	eGFR (mL/min/1.73 m²)	107 (15–164)/109 (13–166)	102.0 ± 30.6/102.1 ± 32.9
	UPCR (g/g)	2.13 (0.2–21.6)/2.76 (0.1–13.3)	3.3 ± 2.7/2.9 ± 2.5
	Anti‐dsDNA > 30 IU/mL, *n*	57/61	42/46
	Low C3 (< 90 mg/dL), *n*	77/76	43/37
	Low C4 (< 16 mg/dL), *n*	32/42	37/44
	Median SLEDAI‐2K score (range)	10 (4–83)/12 (2–35)	Not reported

### 4.3 Risk of Bias Assessment

**FIGURE 2:**
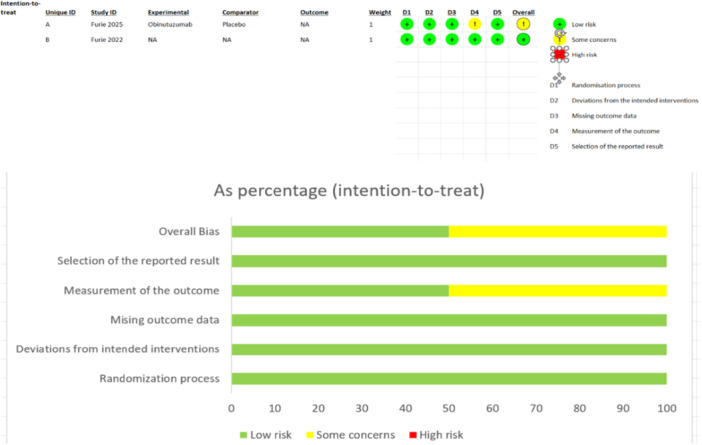
Risk of bias assessment.

Bias was assessed using the RoB 2 tool across five domains. They rated each domain as “low risk,” “some concerns,” or “high risk” of bias (Figure 2). Furie et al. (2022) were judged to have a low risk of bias across all domains, reflecting strong methodological rigor. In contrast, Furie et al. (2025) were rated as having some concerns due to potential bias in Domain 4 (measurement of the outcome), where the outcome assessors may not have been adequately blinded, raising the possibility of detection bias. Nonetheless, both studies were considered methodologically sound for inclusion, with an overall low to moderate risk of bias.

### 4.4 Efficacy Outcomes

#### 4.4.1 Renal Response Outcomes

Obinutuzumab demonstrated clinically meaningful improvements in renal response rates across clinical trials. In the REGENCY trial (2025), patients achieved a 46.4% complete renal response (CRR) at week 76, showing statistical significance compared to standard therapy. The NOBILITY trial (2022) showed a lower initial CRR of 35% at week 52, which improved to 41% by week 104, suggesting a delayed but sustained treatment effect. Notably, in REGENCY, even with prednisone tapering to 7.5 mg/day during weeks 64–76, CRR remained high at 42.7%, highlighting obinutuzumab's potential as a steroid‐sparing agent.

Reduction in proteinuria, a key marker of renal damage, was consistently observed. In REGENCY, 55.5% of patients achieved UPCR < 0.8 without intercurrent events. The NOBILITY trial reported even higher rates over time: 65% at week 52 and 71% at week 104. Additionally, 62% of obinutuzumab‐treated patients in NOBILITY achieved UPCR < 0.5 by week 104 compared to 37% in controls, demonstrating strong long‐term renal protection (Table 2).

**TABLE 2:** Results summary of efficacy outcomes in included trials.

Outcome	REGENCY (furie 2025)	NOBILITY (furie 2022)
Complete renal response (CRR)	46.4% at week 76 (*p* = 0.02)	35% at week 52 (*p* = 0.115), 41% at week 104 (*p* = 0.026)
CRR with prednisone 7.5 mg/day	42.7% at weeks 64–76 (*p* = 0.04)	Not reported
UPCR < 0.8 without intercurrent event	55.5% (*p* = 0.02)	65% at wk 52 (*p* = 0.085), 71% at wk 104 (*p* = 0.003)
UPCR < 0.5	Not reported	52% at wk 52 (*p* = 0.102), 62% at wk 104 (*p* = 0.005)
Change in eGFR (mL/min/1.73 m²)	+2.31 vs. −1.54 (adj diff 3.84)	+9.7 (*p* = 0.017)
Overall renal response (ORR)	59.1% vs. 50.7%	56% at wk 52 (*p* = 0.025), 54% at wk 104 (*p* = 0.005)
Death or renal‐related event	18.9% vs. 35.6% (adj diff −16.8)	5 deaths total: 1 obinutuzumab, 4 placebo
Change in FACIT‐F score	+1.8 vs. +3.1 (adj diff −1.4)	Not reported
Modified CRR (mCRR) at week 52	Not reported	46% vs. 39% (*p* = 0.373)
Modified CRR (mCRR) at week 104	Not reported	56% vs. 34% (*p* = 0.015)
Change in C3 (mean)	Greater increase	30 vs. 12 at wk 52, 29 vs. 11 at wk 104 (*p* < 0.001)
Change in C4 (mean)	Greater increase	9.7 vs. 0.8 at wk 52, 9.6 vs. 0.4 at wk 104 (*p* < 0.001)
Change in anti‐dsDNA (log mean)	Greater decrease	−0.91 vs. −0.10 at wk 52, −1.1 vs. −0.05 at wk 104 (*p* < 0.001)

#### 4.4.2 Renal Function and Overall Response

Renal function, measured by eGFR, improved in both trials. REGENCY showed a 2.31 mL/min/1.73 m² increase vs. a −1.54 decline in controls. Meanwhile, NOBILITY reported a more pronounced +9.7 mL/min/1.73 m² improvement. The overall renal response was 59.1% in REGENCY vs. 50.7% in controls, while NOBILITY reported 56% at week 52 and 54% at week 104, reinforcing obinutuzumab's efficacy (Table 2).

#### 4.4.3 Immunological and Biomarker Effects

Obinutuzumab significantly improved complement levels, indicating reduced disease activity. In NOBILITY, C3 increased by mean value of 30 in the obinutuzumab group vs. 12 in placebo group at week 52, while C4 rose by 9.7 in the obinutuzumab group vs. 0.8 in placebo group. Additionally, anti‐dsDNA antibodies decreased by −0.91 log mean vs. −0.10 at week 52, with further reduction to −1.1 by week 104. These findings suggest robust immunological modulation, correlating with clinical benefits (Table 2).

#### 4.4.4 Long‐Term Outcomes and Safety

In proliferative lupus nephritis, obinutuzumab showed steady and long‐lasting effectiveness. The REGENCY trial (Table 3) reported a significant decrease in the risk of adverse renal events (18.9% rate of death or renal‐related events vs. 35.6% in controls), improvements in proteinuria and eGFR, a steroid‐sparing benefit, and positive immunological effects. The NOBILITY trial recorded only one death in the obinutuzumab group compared to four in the placebo group, but REGENCY reported three deaths in the treatment arm (two from COVID‐19 pneumonia and one from nephrotic syndrome) compared to one COVID‐19 death in the placebo group, indicating a relatively favorable profile in terms of mortality. In REGENCY, patient‐reported fatigue as measured by FACIT‐F showed little variation between groups (1.8 vs. 3.1), while NOBILITY did not measure this. The safety profile was thoroughly assessed in both trials; overall adverse event rates were comparable between treatment groups; however, serious adverse events (SAEs) varied, with NOBILITY reporting lower rates (25% vs. 30%) and REGENCY reporting higher rates (32.4% vs. 18.2%) with obinutuzumab. Infection‐related outcomes also differed; in REGENCY, significant infections were more common with obinutuzumab (16.9% vs. 7.6%, including 15.4% Grade ≥ 3 infections), whereas in NOBILITY, they were less common in the therapy group (8% vs. 18%). Hematologic safety signals included drug‐related neutropenia in 12.5% of obinutuzumab patients in REGENCY, and infusion‐related responses were moderately more frequent with obinutuzumab in both studies. By week 76, all of these reactions had disappeared or were resolving.

**TABLE 3:** Result summary of safety outcomes in the included trials.

Safety outcome	Richard A. furie 2025 (REGENCY)	Richard A. furie 2022 (NOBILITY)
Any adverse event	92.6% (obinutuzumab) vs. 88.6% (placebo)	91% (obinutuzumab) vs. 89% (placebo)
Serious adverse events (SAEs)	32.4% (obinutuzumab) vs. 18.2% (placebo)	25% vs. 30%
Serious infections	16.9% (obinutuzumab) vs. 7.6% (placebo)	8% vs. 18%
Infusion‐related reactions (any grade)	15.4% (obinutuzumab) vs. 11.4% (placebo)	16% (obinutuzumab) vs. 10% (placebo)
Deaths	3 deaths in obinutuzumab group (2 COVID‐19 pneumonia, 1 nephrotic syndrome); 1 in placebo (COVID‐19)	1 death in obinutuzumab group, 4 in placebo group
Grade ≥ 3 infections	15.4% (obinutuzumab) vs. 6.8% (placebo)	Not separately reported
Neutropenia (drug‐related)	Reported more frequently in obinutuzumab group (12.5%); all resolved or resolving by week 76	Not reported

These results show that obinutuzumab is a promising treatment for long‐term lupus nephritis management, considering that it provides significant renal protection and a generally reassuring safety profile. However, the variability in adverse events across studies supports the need for ongoing monitoring.

## 5 Discussion

This systematic review aims to provide a thorough evaluation of the safety and efficacy of obinutuzumab, a type II monoclonal antibody, in treating patients with lupus nephritis (LN).

LN is a frequently occurring severe manifestation of SLE, a complex autoimmune disorder that most commonly affects the kidneys [13]. LN is the main cause of death and morbidity in patients with SLE, and approximately 20% of the individuals advance toward end‐stage renal disease [14]. It arises from the inflammation of the kidney due to the complicated association between adaptive and innate defense reactions. Hereditary, epigenetic, and environmental factors all contribute to the development of LN [15]. The condition can present with multiple symptoms, such as proteinuria, nephritic or nephrotic syndrome, hematuria, renal insufficiency, tubular abnormalities, and, in some cases, hypertension [16]. The therapeutic framework typically includes corticosteroids in combination with CYC or MMF in the induction phase. The maintenance phase constitutes a lower dose of corticosteroid with MMF or AZA [17]. Treatment mainly depends on the histological class of the disease, and currently, there are six distinctive classes of the disease identified on a kidney biopsy [18]. Corticosteroids have become the standard of care (SOC) in the management of LN; however, they are associated with adverse effects, such as hypertension, thromboembolism, menstrual cycle imbalance, edema, gastrointestinal disturbances, fractures, muscle pain, and glaucoma [19, 20].

Obinutuzumab is a type II monoclonal antibody that targets CD20, a surface antigen expressed on B‐cells. It is used primarily in B‐cell malignancies and has shown improved responses in conditions such as chronic lymphocytic leukemia and follicular lymphoma when combined with chemotherapy. It has also been tested for the treatment of LN in placebo‐controlled trials [9, 10], showing better CRR, eGFR, UPCR, and immunological responses.

Our review shows that obinutuzumab demonstrated enhanced CRR consistently compared to placebo when added to the standard treatment plan, as evident in both NOBILITY and REGENCY trials. In particular, the REGENCY and NOBILITY trials reported a CRR of 46.4% at week 76 and 35% at week 52, respectively [9, 10]. These rates significantly surpass the CRR of 10%–40% at 12 months observed with SOC treatments, such as corticosteroids, MMF, CYC, and AZA [19]. Rituximab, a type I anti‐CD20 monoclonal, achieved a CRR of only 26.4% at 52 weeks when tested on LN patients [21]. These results suggest that obinutuzumab may more effectively induce remission in patients with active LN.

One of the biggest therapeutic challenges in treating LN is achieving a long‐term reduction in proteinuria, a critical prognostic indicator of renal survival and disease activity. Decreased proteinuria levels show good responses to the treatment of LN [9, 15]. Both NOBILITY and REGENCY showed sustained reductions in UPCR over the long term, with nearly 65% of the patients showing a UPCR < 0.8 at week 52 and 62% achieving UPCR < 0.5 at 2 years in the NOBILITY trial [9, 10]. This proteinuria‐lowering effect supports its potential as a long‐term disease‐modifying agent.

Additionally, obinutuzumab significantly improved complement levels (C3 and C4) and reduced anti‐dsDNA titers, reinforcing its mechanistic role in modulating the immunopathogenesis of LN. This is important because anti‐dsDNA antibodies are specific to SLE and reflect LN disease activity. They are responsible for many features of the disease, as these antibodies deposit in the kidney and also cause proteinuria [22, 23]. Anti‐dsDNA antibody reduction by obintuzumab reduction implies a good response and can also potentially lead to decreased proteinuria.

Safety outcomes were generally acceptable but require continuous surveillance. Adverse events were frequent in both obinutuzumab and placebo groups (92.6% vs. 88.6%, respectively), reflecting the high‐risk nature of the LN population. Although serious infections were more common in the obinutuzumab arm of REGENCY, this pattern was not replicated in NOBILITY. Infusion reactions and neutropenia were reported but were manageable in most cases. Importantly, mortality rates were not elevated with obinutuzumab.

Although no head‐to‐head trials comparing obinutuzumab and rituximab in lupus nephritis have been conducted, these biologics differ in their mechanisms and clinical effects. Rituximab, a type I anti‐CD20 monoclonal antibody, relies predominantly on complement‐dependent cytotoxicity and ADCC for B‐cell depletion. Obinutuzumab, a type II anti‐CD20 antibody, induces enhanced direct cell death and stronger ADCC, potentially achieving more complete and sustained B‐cell depletion. Indirect comparisons suggest higher complete renal response rates with obinutuzumab in the NOBILITY and REGENCY trials, along with potential steroid‐sparing benefits. Safety profiles are generally comparable, though mechanistic differences may influence infusion reactions. Future direct comparative studies are warranted to validate these observations [9, 10, 24].

These results support the therapeutic potential of obinutuzumab in LN. However, several limitations must be considered. First, A major limitation of this review is the inclusion of only two randomized controlled trials. The small number of studies reduces the precision of pooled estimates and limits the reliability of heterogeneity assessment. Additionally, formal evaluation of publication bias is not feasible with such a small evidence base [25]. Consequently, results should be interpreted with caution. Although both trials were methodologically robust and demonstrated consistent efficacy trends, further large‐scale randomized studies are required to confirm long‐term safety and effectiveness. Second, while efficacy outcomes were promising, the trials had relatively short follow‐up durations to assess long‐term renal survival or progression to end‐stage renal disease (ESRD). Additionally, variability in patient characteristics and treatment protocols between trials may contribute to heterogeneity in outcomes. Future research should prioritize large‐scale, multicenter trials with diverse populations to confirm long‐term safety and efficacy. Comparative trials directly evaluating obinutuzumab against other biologics, such as belimumab or rituximab, could clarify its positioning within LN treatment.

## 6 Conclusion

Current evidence indicates that obinutuzumab is a novel and potentially transformative therapy for proliferative lupus nephritis. It shows superiority in achieving renal response, reducing proteinuria, and improving immunological profiles, with an acceptable safety profile. Its steroid‐sparing potential and ability to delay renal deterioration make it a compelling candidate for broader clinical use, pending further validation.

## Author Contributions

**Zainab Arif:** conceptualization, writing – original draft, writing – review and editing. **Hafsa Ali:** writing – original draft, writing – review and editing. **Erum Siddiqui:** writing – original draft, writing – review and editing. **Shorrem Naeem:** writing – original draft, writing – review and editing. **Mohammed Hammad Jaber Amin:** writing – review and editing.

## Ethics Statement

The authors have nothing to report.

## Consent

The authors have nothing to report.

## Conflicts of Interest

The authors declare no conflicts of interest.

## Policy on Using ChatGPT and Similar AI Tools

The authors declare that no artificial intelligence (AI) or AI‐assisted technologies were used in the writing or editing of this manuscript.

## Data Availability

Data openly available in a public repository that issues datasets with DOIs (This systematic review was registered with PROSPERO 2025 CRD420251074418, Available from https://www.crd.york.ac.uk/PROSPERO/view/CRD420251074418).
